# Mild Hyperglycaemia in Hospitalised Children with Moderate COVID-19 Infection

**DOI:** 10.3390/medicina59050944

**Published:** 2023-05-14

**Authors:** Jarmila Vojtková, Peter Bánovčin, Anna Ďurdíková, Elena Nováková, Miloš Jeseňák

**Affiliations:** 1 Department of Paediatrics, Comenius University in Bratislava, Jessenius Faculty of Medicine and University Hospital, 036 01 Martin, Slovakia; 2 Department of Microbiology and Immunology, Comenius University in Bratislava, Jessenius Faculty of Medicine, 036 01 Martin, Slovakia; 3 Department of Clinical Immunology and Allergology, University Hospital in Martin, 036 01 Martin, Slovakia; 4 Department of Pulmonology and Phthisiology, Comenius University in Bratislava, Jessenius Faculty of Medicine and University Hospital, 036 01 Martin, Slovakia

**Keywords:** COVID-19, SARS-CoV2, RNA virus infection, hyperglycaemia, gastrointestinal symptoms, fever

## Abstract

*Background and Objectives*: COVID-19 infection may influence many physiological processes, including glucose metabolism. Acute hyperglycaemia has been related to a worse prognosis in patients with severe COVID-19 infection. The aim of our study was to find out if moderate COVID-19 infection is associated with hyperglycaemia. *Materials and Methods*: A total of 235 children were enrolled in the study between October 2021 and October 2022, 112 with confirmed COVID-19 infection and 123 with other RNA viral infection. In all patients, types of symptoms, glycaemia at the time of admission, and basic anthropometric and biochemical parameters were recorded. *Results*: Average glycaemia was significantly higher in COVID-19 patients compared to other viral infections (5.7 ± 1.12 vs. 5.31 ± 1.4 mmol/L, *p* = 0.011). This difference was more obvious in subgroups with gastrointestinal manifestations (5.6 ± 1.11 vs. 4.81 ± 1.38 mmol/L, *p* = 0.0006) and with fever (5.76±1.22 vs. 5.11±1.37 mmol/L, *p* = 0.002), while no significant difference was found in subgroups with mainly respiratory symptoms. The risk of hyperglycaemia (>5.6 mmol/L) was higher in COVID-19 patients compared to other viral infections (OR = 1.86, 95%CI = 1.10–3.14, *p* = 0.02). The risk of hyperglycaemia was significantly higher in COVID-19 compared to other viral infections in the subgroups of patients with fever (OR = 3.59, 95% CI 1.755–7.345, *p* = 0.0005) and with gastrointestinal manifestations (OR = 2.48, 95% CI 1.058–5.791, *p* = 0.036). *Conclusion*: According to our results, mild hyperglycaemia was significantly more common in children with moderate COVID-19 infection compared to other RNA virus respiratory and gastrointestinal infections, especially when accompanied by fever or gastrointestinal symptoms.

## 1. Introduction

Stress hyperglycaemia may accompany acute conditions (infections, burns, ischaemia, and others) as a possible mechanism of adaptation to disease and has been linked to poor outcomes [1]. Its frequency varies in various types of acute condition [2,3,4,5] and may be related to disease severity, comorbidities, or medication used. Its complex aetiology comprises many factors, such as increased formation of reactive oxygen species with mitochondrial dysfunction, increased formation of inflammatory cytokines, and stimulation of contraregulatory hormones (cortisol, epinephrine) [6,7,8,9]. These factors contribute to increased liver gluconeogenesis, peripheral insulin resistance, and beta cell dysfunction [7]. Within the illness, gluconeogenesis is induced mainly by glucagon, along with the contribution of epinephrine and cortisol. Moreover, insulin is unable to inhibit liver gluconeogenesis due to insulin resistance. Peripheral insulin resistance is a consequence of dysfunction in post-receptor insulin signalling and the down-regulation of glucose transporter 4 [10]. Stress hyperglycaemia might be not considered as diabetes mellitus [11]; however, whether it could be a risk factor for further diabetes development has not yet been fully clarified.

The relationship between COVID-19 infection and hyperglycaemia is bidirectional [12]. On the one hand, hyperglycaemia and diabetes mellitus have been shown to be risk factors for higher morbidity and mortality in patients with COVID-19 [13,14]. On the other hand, COVID-19 contributes to hyperglycaemia (and even diabetes) due to impaired pancreatic beta-cell function and cytokine storm [15]. 

Several studies and reports have indicated that severe hyperglycaemia and diabetes mellitus are associated with higher mortality and poor prognosis in COVID-19 patients [16,17]. There is a small amount of data on how mild COVID-19 infection influences glucose level [18,19], and all was obtained from the adult population. Therefore, we aimed to evaluate the association between glycaemia and selected clinical and laboratory features of mild COVID-19 infection and to compare it with other RNA viral respiratory infections. 

## 2. Materials and Methods

### 2.1. Subjects

In this retrospective single-centre study, we collected data from medical records of children hospitalised in the Department of Paediatrics at the University Hospital, Martin, Slovakia, between October 2021 and October 2022. The study group consisted of children with COVID-19 infection confirmed by real-time PCR tests for SARS-CoV-2, retrieved from nose and throat swabs. We documented the presence of fever and manifestation of COVID-19 infection—respiratory (acute laryngitis, rhinopharyngitis, bronchitis, cough), gastrointestinal (poor feeding, vomiting, nausea, diarrhoea), both, or other (hypotension, hypertension, myalgia, arthritis). 

The control group consisted of children hospitalised in the same department during the same period with RNA viral infection with either gastrointestinal manifestations (gastritis, gastroenteritis or enteritis) or respiratory manifestations (acute laryngitis or acute obstructive bronchitis). Viruses were confirmed via real-time PCR tests—rotavirus, norovirus for gastrointestinal manifestations and respiratory syncytial virus (RSV), or rhinovirus for respiratory manifestations. All patients in the control group had a negative PCR test for SARS-CoV-2. 

The exclusion criteria for patients were known diabetes mellitus or prediabetes, severe kidney disease, severe neurological or cardiological disease (hydrocephalus, cardiomyopathy), use of corticoids in any form (inhalation, peroral, rectal, intravenous, intramuscular) in the last four weeks, use of any drugs that could potentially increase glucose levels (beta blockers, beta mimetics, immunosuppressants) in the last four weeks, acid-base or mineral imbalance (pH ≤ 7.3, bicarbonates ≤ 15 mmol/L, sodium, potassium and chlorides not in normal range), severe infection requiring admission to the paediatric intensive care unit, and multisystem inflammatory syndrome in children. Patients with confirmed bacterial infection (CRP ≥ 20 mg/L, positive bacterial swabs), with DNA virus infection (e.g., adenovirus), or with more than one viral infection (e.g., rotavirus at the same time as SARS-CoV-2) were not enrolled in the study. 

### 2.2. Followed Parameters

In both groups, basic anthropometric parameters (age, sex, weight), the length of hospitalisation, the number and time of vaccinations against SARS-CoV-2, and biochemical and haematological parameters (glycaemia, minerals, pH, differential blood count) were followed. Prevailing symptoms (respiratory or gastrointestinal) and the presence of fever were recorded in both groups. Glycaemia ≥5.6 mmol/L was considered as mild hyperglycaemia, glycaemia ≥7.8 mmol/L as moderate hyperglycaemia, and glycaemia ≥11.1 mmol/L as severe hyperglycaemia in accordance with the definition of the limit for normal glycaemia, prediabetes, and diabetes mellitus [11]. 

### 2.3. Statistics 

The results were statistically processed using the statistical program SYSTAT 11. A *p*-value less than 0.05 was considered as statistically significant and the Benjamini–Hochberg method was used to adjust *p*-value [20]. To assess the risk of specific parameters, the odds ratio (OR) and 95% confidence interval (95% CI) were calculated, while an univariate model and age and sex-adjusted models were also used. The Pearson correlation test was used to establish the correlation between two variables: r ≤ 0.3 was considered a weak correlation, r = 0.31–0.69 was considered a moderate correlation, and r ≥ 0.7 was considered a strong correlation. 

## 3. Results

### 3.1. Characteristics of Enrolled Patients

A total of 235 paediatric patients aged 0.1–18 years were enrolled into the study, 112 with confirmed COVID-19 infection and 123 with another confirmed RNA virus (rotavirus, norovirus, RSV or rhinovirus). Symptoms requiring hospitalisation in patients with COVID-19 infection were respiratory symptoms in 69 patients (61.61%), gastrointestinal symptoms in 41 subjects (36.61%), both respiratory and gastrointestinal symptoms in 14 patients (12.5%), fever in 81 subjects (72.32%), and other symptoms (hypotension, hypertension, hypothermia, myalgia) in 8 patients (7.14%). 

In the group with other RNA virus infections, 65 patients had a respiratory infection (52.84%) and 58 subjects had a gastrointestinal infection (47.15%), while fever (defined as ≥38.5 °C) was found in 73 patients (59.35%). The characteristics of enrolled subjects are shown in Table 1. 

In the COVID-19 group, 2 patients (15 and18 years old) were vaccinated with the first dose of nucleoside-modified mRNA vaccine against SARS-CoV-2 (7 and 12 days before hospitalisation), and none of the patients were fully vaccinated. In the group of other RNA virus infection, 4 patients were fully vaccinated against SARS-CoV-2, and 1 patient received the first dose of nucleoside-modified mRNA vaccine 8 days before hospitalisation (all mentioned children at the age 11–18 years).

### 3.2. Glycaemia in Various Subgroups

**Figure 1:**
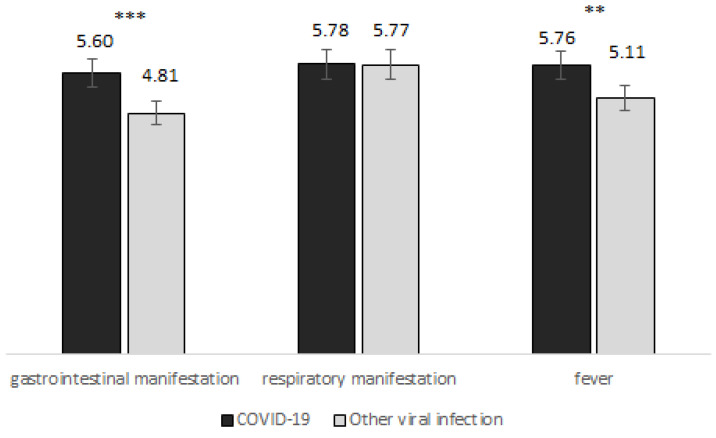
Average glycemia in patients with COVID-19 and other viral infections divided according to the symptoms (** *p* < 0.01; *** *p* < 0.001).

Average glycaemia was higher in COVID-19 patients compared to subjects with other viral infections (5.7 ± 1.12 vs. 5.31 ± 1.4 mmol/L, *p* = 0.011, adjusted *p* = 0.095). This difference was more obvious in subgroups with gastrointestinal manifestations (5.6 ± 1.11 mmol/L in COVID-19 patients compared to 4.81 ± 1.38 mmol/L in other viral infections, *p* = 0.0006, adjusted *p* = 0.010), while no significant difference was found in subgroups with mainly respiratory symptoms (Figure 1). When dividing patients according to the presence of fever, glycaemia was significantly higher in COVID-19 patients compared to patients with other viral infections (5.76 ± 1.22 vs. 5.11 ± 1.37 mmol/L, *p* = 0.002, adjusted *p* = 0.034) (Table 2). 

Mild hyperglycaemia ≥5.6 mmol/L was found in 55 patients (49.1%) in the COVID-19 group compared to 42 patients (34.14%) with other viral infections (OR = 1.86, 95%CI = 1.10–3.14, *p* = 0.02). Hyperglycaemia ≥7.8 mmol/L was found in 6 patients (5.35%) with COVID-19 infection and in 7 patients (5.69%) with other viral infections, while no patient had hyperglycaemia ≥11.1 mmol/L. Hypoglycaemia <3.5 mmol/L was found in 1 patient with COVID-19 infection and in 9 patients in the control group (all with gastrointestinal manifestations) (OR = 0.114, 95% CI 0.014–0.916, *p* = 0.041), while no significant difference was found in minerals or acid-base level. In the subgroup of patients with gastrointestinal manifestations, hyperglycaemia (≥5.6 mmol/L) was found in 46.34% of COVID-19 patients compared to 25.86% of patients with other viral infections (OR = 2.48, 95% CI 1.058–5.791, *p* = 0.036 in univariate model). In the subgroup of patients with fever, hyperglycaemia was found in 56.52% of COVID-19 patients compared to 23.07% of patients with other viral infections (OR = 3.59, 95% CI 1.755–7.345, *p* = 0.0005 in univariate model, OR = 2.18, 95% CI 1.095–4.564, *p* = 0.012 in age and sex-adjusted model) (Table 3). 

### 3.3. Correlations

Correlations between glycaemia and other followed parameters are shown in Table 4. In patients with COVID-19, a moderate positive correlation was found between glycaemia and the percentage of neutrophils (r = 0.32, *p* = 0.005), and a weak positive correlation was found between glycemia and CRP (r = 0.254, *p* = 0.034) and total leucocytes (r = 0.284, *p* = 0.016). Moreover, a negative moderate correlation was found between glycaemia and potassium levels (r = −0.31, *p* = 0.008) and the percentage of lymphocytes (r = −0.328, *p* = 0.004). In patients with other viral infections, a moderate positive correlation was found between glycaemia and total leucocytes (r = 0.33, *p* = 0.003), total neutrophils (r = 0.31, *p* = 0.008), and total eosinophils (r = 0.35, *p* = 0.001).

## 4. Discussion

According to our results, average glycaemia was higher in COVID-19 patients compared to other RNA virus infections, and this difference was more obvious in subgroups with gastrointestinal manifestations and with fever, while no significant difference was found in subgroups with mainly respiratory symptoms. Patients with COVID-19 had an almost two-fold higher risk of hyperglycaemia (>5.6 mmol/L) compared to other viral infections, especially in the subgroups of patients with fever and with gastrointestinal manifestations. 

COVID-19 infection has been shown to influence many organ systems, including the endocrine system and metabolism [21]. COVID-19 infection may be accompanied by stress hyperglycaemia [16], and impaired glucometabolic control has been found even after recovery from COVID-19 [22]. 

Most papers describing an association between COVID-19 and hyperglycaemia were focused on critically ill patients hospitalised in an intensive care unit (ICU) [16,17]. Our work deals with the mild form of COVID-19 infection in patients hospitalised in a paediatric ward not requiring treatment in the ICU. This is probably why no severe hyperglycaemia (≥11.1 mmol/L) was observed and only about 5% of patients had hyperglycaemia ≥7.8 mmol/L. According to our results, COVID-19 infection was associated with an almost two-fold higher risk of mild hyperglycaemia (>5.6 mmol/L) compared to other RNA infections. 

RNA viruses (including SARS-CoV-2) influence the host cellular metabolism in many ways [23]. They up-regulate glycolysis and glycogenolysis and induce the anabolic reprogramming of the host cell metabolism via overexpression of the GLUT1 receptor, leading to increased glucose uptake and increased intermediates in the pentose pathway. Numerous viruses induce glutaminolysis and fatty acid synthesis [23]. These alterations of carbohydrate metabolism in infected cells can provide cellular substrates for viral particles and energy for viral replication. The relationship between viruses and hyperglycaemia (even increased risk of type 1 diabetes) may also involve the destruction of pancreatic beta cells. Viruses attract natural killer cells and T cells that produce cytokines (TNF-α, IFN-γ, IL-1β), resulting in damage to pancreatic beta cells. The incidence of type 1 diabetes (T1D) may be accelerated due to molecular mimicry if viral antigens have the homology of beta-cell epitopes [24]. 

COVID-19 infection is particular in many ways. Islet beta cells present ACE-2 receptors to which the SARS-CoV2 spike protein may bind, causing subsequent beta cell infection and an inflammatory response [24]. It seems that the global COVID-19 pandemic is associated with a higher incidence of type 1 diabetes and diabetic ketoacidosis [25] and even with a higher severity of diabetic ketoacidosis [26]. When compared to the situation before the COVID-19 pandemic, children with new-onset T1D had higher hyperglycaemia and glycosylated hemoglobin (HbA1c) [23] and a higher insulin requirement [27]. 

COVID-19 infection is associated with a specific immune response [28] and higher production of cytokines (TNF-α, IL-1, IL-6, interferon-γ and IL-17A) [29]. These elevated cytokines (or cytokine storm in a severe case of COVID-19) may interfere with the insulin signalling pathway, reduce insulin production, and increase blood glucose. This may explain why the condition of fever during COVID-19 infection was associated with a 3.5-times higher risk of mild hyperglycaemia compared to fever during other RNA virus infections, according to our results. 

It has been reported that the death rate for COVID-19 is significantly higher than that of influenza [30]. Compared to adults, COVID-19 in childhood has a usually milder course, which may be explained by few factors, such as lower affinity of ACE-receptor to SARS-CoV2, lower expression of ACE-2 gene in nasal epithelium, different interferon and T cell response, lower prevalence of risk comorbidities, protective heterologous effect of live vaccines, and “trained” innate immunity due to higher exposure of respiratory viruses in childhood [29]. Severe cases of COVID-19 have been rarely reported in childhood and may be related to comorbidities such as immunosuppression, obesity, chronic pulmonary disease, cardiovascular disease, neuromuscular, and neurodevelopmental disease [10]. In our study, only patients with a moderate course of COVID-19 and 100% survival were enrolled. Additionally, in the subgroup of patients with other RNA virus infections, all children recovered within a few days, and the overall survival was 100%. In reported papers dealing with severe cases of COVID-19, more severe glycaemia may be considered as a risk factor of a poorer prognosis. Thus, more severe glycaemia induced by COVID-19 might be due to more aggressive behaviour of SARS-CoV2 virus compared to other RNA viruses. Another possibility is that SARS-CoV2 might have an affinity to more tissues and influence many organ systems, including those involved in glucose metabolism, so stress hyperglycaemia may occur more frequently.

CRP is considered as a marker of tissue injury and inflammation. It has been reported that COVID-19 infection is also associated with an increase in CRP level [31]. In our study, no significant difference was found in any subgroups when comparing CRP levels. This may be caused by the mild course of COVID-19, low number of enrolled subjects, and by the fact that children with a higher CRP level were not included in the study. In the COVID-19 group, a weak significant correlation was found between CRP level and glucose. This might be explained by the possibility that CRP and glucose are increased due to tissue damage caused by SARS-CoV2, which could be more severe than in other RNA virus infections.

In our study, the combination of gastrointestinal symptoms with COVID-19 infection was associated with a 2.5-times higher risk of hyperglycaemia compared to other viral infections. One of the possible explanations may be the fact that rotavirus infection has a more severe course than COVID-19 regarding vomiting and oral intolerance [32], so the tendency for hypoglycaemia may be higher. To avoid biased results, all patients with acidosis and mineral imbalance were excluded from the study. This finding may be clinically useful in the differential diagnosis of patients admitted to hospital with emesis or diarrhoea. The process from admission until confirmation of diagnosis may take some time, and patients with hyperglycaemia may be more likely to have COVID-19 infection, thus necessitating isolation. 

The rate of vaccination against SARS-CoV2 is less frequent in the paediatric population when compared to adults. Data in the United States claim that 2% of children younger than 2 years, 4% of children at the age 2–4 years, 32% of children aged 5–11 years, and 61% of children at the age 12–17 years are fully vaccinated compared to 93% of adults older than 65 years [33]. This may be explain the mild cases of the disease in childhood, lower mortality (1 per million in children compared to 6500 per million in patients older than 80 years), and parents‘ lack of fear regarding infection. In our study, four children were fully vaccinated with a nucleoside-modified mRNA vaccine, and none of them tested positive for COVID-19. Three children were vaccinated with the first dose of the vaccine, and two of them tested positive for COVID-19. In all likelihood, they were probably in the incubation period of infection at the time of their vaccination. The majority of enrolled children were in the youngest age (up to 5 years), and parents hesitated about vaccine necessity and safety despite the fact that side effects are only rare [34]. COVID-19 in children is either asymptomatic or with mild to moderate symptoms, and the mortality rate is low. Although, COVID-19 is far less devastating in children compared to adults, children and adolescents are at risk of experiencing serious complications such as prolonged clinical symptoms (“long COVID-19”) or hyperinflammatory syndrome after COVID-19 (multisystem inflammatory syndrome in children, MIS-C). Vaccination against SARS-CoV2 is beneficial in the prevention of these complications too.

The limitations of this study include the relatively small number of enrolled subjects, the lack of a control group of healthy children, its retrospective character, and limited number of parameters followed. We only followed glycaemia at admission, and not the stress hyperglycaemia ratio (ratio of glycated haemoglobin to glycaemia), as glycated haemoglobin is not routinely followed at the time of admission of common acute diagnoses. Similarly, it would be useful to examine the variants of SARS-CoV2, subtypes of lymphocytes, and specific cytokine levels. For further follow-up, it would be beneficial to identify the possible development of prediabetes or diabetes mellitus or the speed of recovery after suffering from normoglycaemia. 

## 5. Conclusions

To the best of our knowledge, this is the first study describing an association between hyperglycaemia at the time of admission to hospital and COVID-19 infection with mild manifestations in children. To summarise, moderate COVID-19 infection (especially with fever and gastrointestinal manifestations) is more strongly associated with mild hyperglycaemia than other RNA virus infections of the respiratory or gastrointestinal tract. 

## Figures and Tables

**Table 1:** Characteristics of study and control group.

	COVID-19 (*n* = 112)	Other RNA Virus Infection (*n* = 123)	Raw *p*	Adjusted *p*
Age	3.16 ± 2.87	3.37 ± 2.9	0.214	0.294
Sex	56 females/56 males	59 females/64 males	0.384	0.402
Days of hospitalisation	3.14 ± 2.69	2.91 ±1.8	0.041	0.180
Fever	81 (72.32%)	73 (59.35%)	0.172	0.271
Mainly respiratory symptoms	69 (61.61%)	65 (52.84%)	0.267	0.314
Mainly gastrointestinal symptoms	41 (36.61%)	58 (47.15%)	0.145	0.261
Glucose (mmol/L)	5.7 ± 1.12	5.31 ± 1.4	0.011	0.095
Sodium (mmol/L)	136.30 ± 2.18	135.35 ± 2.42	0.092	0.184
Potassium (mmol/L)	4.39 ± 0.56	4.35 ± 0.54	0.273	0.314
Chloride (mmol/L)	104.01 ± 2.92	103.11 ± 2.64	0.074	0.184
Bicarbonates (mmol/L)	20.03 ± 2.97	19.23 ± 2.32	0.087	0.184
pH	7.44 ± 0.04	7.42 ± 0.05	0.064	0.181
CRP (mg/L)	3.74 ± 3.87	4.32 ± 3.14	0.285	0.314
Leukocytes (×10^9^/L)	9.12 ± 4.78	10.42 ± 4.11	0.013	0.095
Neutrophils (×10^9^/L)	5.16 ± 4.17	6.16 ± 4.11	0.037	0.180
Neutrophils (%)	54.17 ± 25.15	56.15 ± 25.18	0.276	0.314
Lymphocytes (×10^9^/L)	2.78 ± 2.35	3.12 ± 2.62	0.156	0.261
Lymphocytes (%)	31.97 ± 22.01	31.66 ± 23.31	0.457	0.457
Monocytes (×10^9^/L)	0.99 ± 0.68	1.07 ± 0.52	0.193	0.283
Monocytes (%)	12.27 ± 7.93	10.99 ± 4.91	0.068	0.181
Eosinophils (×10^9^/L)	0.08 ± 0.11	0.04 ± 0.09	0.058	0.181
Eosinophils (%)	1.16 ± 1.47	0.39 ± 0.81	0.011	0.095

**Table 2:** Followed parameters in subgroups regarding symptoms compared between patients with COVID-19 and other RNA virus infections.

	Gastrointestinal Manifestation				Respiratory Manifestation				Fever			
	COVID-19 (*n* = 41)	Other Viral Infection (*n* = 58)	*p*	Adjusted *p*	COVID-19 (*n* = 69)	Other Viral Infection (*n* = 65)	*p*	Adjusted *p*	COVID-19 (*n* = 81)	Other Viral Infection (*n* = 73)	*p*	Adjusted *p*
Days of hospitalisation	2.82 ± 1.23	2.23 ± 0.81	0.05	0.100	3.35 ± 2.92	3.46 ± 2.22	0.468	0.485	3.01 ± 1.59	2.67 ± 1.41	0.075	0.232
Glucose (mmol/L)	5.6 ± 1.11	4.81 ± 1.38	0.0006	0.010	5.73 ± 1.21	5.77 ± 1.24	0.386	0.437	5.76 ± 1.22	5.11 ± 1.38	0.002	0.034
Natrium (mmol/L)	136.24 ± 2.18	135.72 ± 2.86	0.056	0.100	136.21 ± 2.72	135.94 ± 1.77	0.101	0.172	136.01 ± 2.01	135.88 ± 2.74	0.078	0.232
Kalium (mmol/L)	4.29 ± 0.57	4.15 ± 0.41	0.07	0.108	4.46 ± 0.58	4.54 ± 0.59	0.184	0.261	4.39 ± 0.54	4.31 ± 0.38	0.184	0.375
Chloride (mmol/L)	103.07 ± 2.93	101.83 ± 2.96	0.059	0.100	103.82 ± 2.76	103.97 ± 1.8	0.485	0.485	103.98 ± 2.93	102.92 ± 2.89	0.062	0.232
Bicarbonates (mmol/L)	21.01 ± 2.34	20.56 ± 2.41	0.092	0.120	22.46 ± 2.22	22.13 ± 2.56	0.234	0.306	21.86 ± 2.34	22.15 ± 2.52	0.285	0.375
pH	7.44 ± 0.05	7.42 ± 0.06	0.099	0.120	7.45 ± 0.05	7.42 ± 0.04	0.085	0.161	7.45 ± 0.05	7.42 ± 0.06	0.079	0.232
CRP (mg/L)	3.66 ± 3.88	4.07 ± 2.98	0.369	0.369	3.99 ± 3.61	4.94 ± 3.68	0.167	0.258	4.01 ± 3.95	4.68 ± 3.22	0.286	0.375
Leukocytes (×10^9^/L)	9.36 ± 4.78	9.75 ± 4.36	0.342	0.363	9.11 ± 4.39	11.03 ± 3.79	0.011	0.051	9.21 ± 4.99	9.37 ± 4.03	0.421	0.447
Neutrophils (×10^9^/L)	5.54 ± 4.17	7.07 ± 4.32	0.051	0.100	5.05 ± 3.89	5.34 ± 3.76	0.298	0.362	5.36 ± 4.31	5.78 ± 4.05	0.293	0.375
Neutrophils (%)	57.78 ± 25.16	67.68 ± 20.7	0.022	0.075	52.86 ± 24.79	45.52 ± 24.39	0.047	0.147	55.61 ± 24.35	57.44 ± 21.73	0.331	0.375
Lymphocytes (×10^9^/L)	2.62 ± 2.35	1.72 ± 1.47	0.018	0.075	2.87 ± 2.26	4.42 ± 2.8	0.011	0.051	2.69 ± 2.32	2.49 ± 1.66	0.289	0.375
Lymphocytes (%)	28.94 ± 22.01	21.44 ± 18.03	0.039	0.075	32.79 ± 21.45	41.08 ± 23.73	0.012	0.051	30.78 ± 21.54	29.11 ± 19.27	0.328	0.375
Monocytes (×10^9^/L)	0.96 ± 0.68	0.91 ± 0.43	0.292	0.331	1.06 ± 0.72	1.22 ± 0.54	0.052	0.147	1.03 ± 0.75	1.09 ± 0.53	0.307	0.375
Monocytes (%)	11.82 ± 7.93	10.14 ± 4.58	0.092	0.120	12.97 ± 8.59	11.78 ± 5.14	0.065	0.157	12.51 ± 8.32	12.45 ± 4.88	0.482	0.482
Eosinophils (×10^9^/L)	0.08 ± 0.97	0.02 ± 0.05	0.013	0.074	0.08 ± 0.99	0.09 ± 0.14	0.078	0.161	0.05 ± 0.75	0.03 ± 0.06	0.082	0.232
Eosinophils (%)	0.95 ± 1.47	0.3 ± 0.84	0.018	0.074	1.31 ± 1.52	0.63 ± 0.74	0.01	0.051	0.67 ± 0.98	0.36 ± 0.54	0.159	0.375

**Table 3:** The risk of hyperglycaemia in various subgroups of patients with COVID-19 and other viral infection.

	Hyperglycaemia in COVID-19	Hyperglycaemia in other Viral Infection	Univariate Model			Age, Sex-Adjusted Model		
			OR	95%CI	*p*	OR	95%CI	*p*
Whole group disregarding symptoms	49.1%	34.14%	1.86	1.10–3.14	0.02	1.12	0.893–2.894	0.094
Subgroup with gastrointestinal manifestation	46.34%	25.86%	2.48	1.058–5.791	0.036	1.97	0.951–3.842	0.061
Subgroup with respiratory manifestation	52.17%	43.07%	1.44	0.729–2.849	0.293	0.95	0.432–2.591	0.384
Subgroup with fever	48.14%	20.54%	3.59	1.755–7.345	0.0005	2.18	1.095–4.564	0.012

**Table 4:** Correlation between glucose and other variables in patients with COVID-19 and other RNA virus infection.

	Glucose (mmol/L) in COVID-19 r	Glucose (mmol/L) in other RNA Virus Infection r
Total leucocytes (×10^9^/L)	0.125	0.331 **
Total neutrophils (×10^9^/L)	0.284 *	0.318 **
Neutrophils%	0.321 **	0.129
Total lymphocytes (×10^9^/L)	−0.233 *	−0.017
Lymphocytes%	−0.328 **	−0.122
Total monocytes (×10^9^/L)	−0.047	0.073
Monocytes%	−0.067	−0.177
Total eosinophils (×10^9^/L)	−0.169	0.353 **
Eosinophils%	−0.146	0.179
Natrium (mmol/L)	0.143	0.173
Potassium (mmol/L)	−0.311 **	−0.102
Chloride (mmol/L)	0.089	0.145
Bicarbonate (mmol/L)	0.095	0.109
pH	−0.081	0.114
CRP (mg/L)	0.254 *	−0.023

* *p* ≤ 0.05, ** *p* ≤ 0.01.

## Data Availability

Data supporting the reported results can be provided if needed or requested by the reviewer.
